# Hydrological Seasonality Drives DOM–Bacteria Interactions in the Rushan River Basin

**DOI:** 10.3390/microorganisms14010110

**Published:** 2026-01-05

**Authors:** Shanshan Zheng, Fan Feng, Dongping Liu, Feng Qian, Xiaolin Xie, Huibin Yu, Yonghui Song

**Affiliations:** 1 State Key Laboratory of Environmental Criteria and Risk Assessment, Chinese Research Academy of Environmental Sciences, Beijing 100012, China; m18661373270@163.com (S.Z.); qbunny1634@163.com (F.F.);; 2 College of Environment, Liaoning University, Shenyang 110036, China

**Keywords:** dissolved organic matter, hydrological seasonality, humification, microbial diversity, co-occurrence network

## Abstract

To unravel hydrological controls on dissolved organic matter (DOM)–microbe interactions in river ecosystems, this study integrated 3D excitation–emission matrix spectroscopy (3D-EEMs), parallel factor analysis (PARAFAC), and 16S rRNA sequencing to characterize seasonal DOM dynamics and microbial assembly in China’s Rushan River Basin. PARAFAC resolved contrasting DOM signatures between dry (four protein-like, two humic-like components) and wet seasons (three protein-like, three humic-like components). Dry-season DOM was dominated by tyrosine-like substances (58.03%), reflecting microbial degradation and phytoplankton activity, while wet-season DOM showed elevated tryptophan-like components (34.38%) and terrestrial fulvic acids (17.14%), which may be related to rain-driven external inputs. The α -diversity of the microbiota is relatively high in the wet season, mainly consisting of *Proteobacteria* (34.06–68.10%) and *Actinobacteriota* (9.15–20.76%). In the dry season community, there are *Bacteroidota* (14.71–38.45%) and *Verrucomicrobiota* (6.13–14.32%). The structural equation model (SEM) semi-quantified the comprehensive pathways by which microorganisms inhibit unstable proteins and enhance humification. These results reveal the synergistic regulatory role of hydrological seasonality on DOM and microorganisms, and provide a basis for adaptive water quality management.

## 1. Introduction

Urban river ecosystems face serious threats from non-point source (NPS) pollution, characterized by its dispersity, uncertainty, and delayed impacts, making prediction and management particularly challenging [1]. As critical receptors of NPS pollutants, urban rivers exhibit water quality dynamics heavily influenced by land use patterns and anthropogenic activities [2], with dissolved organic matter (DOM) serving as a key mediator in biogeochemical cycles [3,4]. DOM, a heterogeneous mixture of organic compounds, plays dual roles in aquatic systems: it acts as a carbon and nutrient source for microbial communities while influencing the fate of pollutants such as heavy metals, nitrogen, and phosphorus [5,6,7]. Despite its ecological significance, the interplay between DOM composition, hydrological variability, and bacterial communities remains poorly understood, particularly in rivers impacted by agricultural and urban runoff.

DOM originates from both autochthonous (e.g., microbial/algal production) and allochthonous sources (e.g., terrestrial plant leaching, wastewater inputs) [8,9]. Hydrological regimes, such as seasonal rainfall and drought, significantly alter DOM inputs and transformation pathways [10,11]. For instance, wet seasons enhance terrestrial DOM influx via surface runoff, while dry seasons may favor microbial reprocessing of endogenous DOM [12]. Advanced techniques like three-dimensional excitation-emission matrix spectroscopy (3D-EEMs) coupled with parallel factor analysis (PARAFAC) have enabled nuanced characterization of DOM fluorescence components, linking them to microbial activity and pollution sources [13,14]. Concurrently, high-throughput sequencing of 16S rRNA has revealed microbial community responses to DOM dynamics [15,16], yet few studies integrate these approaches to unravel DOM–microbe interactions under contrasting hydrological scenarios.

The Rushan River Basin, a typical NPS-polluted system in eastern China, exemplifies these challenges. Dominated by agriculture and livestock farming, the basin receives substantial nutrient and contaminant loads, altering DOM composition and microbial ecology. While prior studies have examined DOM or microbial communities in isolation, the synergistic effects of hydrological variability on DOM–microbe co-evolution remain unexplored. For instance, how microbial taxa mediate DOM humification or degrade protein-like substances during drought versus flood conditions is unclear. Addressing these knowledge gaps is critical for predicting ecosystem responses to climate-driven hydrological shifts and informing targeted river management.

The Rushan River Basin, a critical passage between terrestrial ecosystems and the Yellow Sea coastal zone, serves as a sentinel site for studying land–sea interactions in anthropogenically impacted estuaries. As one of the primary freshwater contributors to the southern Yellow Sea’s nearshore environment, this basin’s DOM–microbe dynamics directly influence coastal carbon cycling and eutrophication processes. This study investigates the composition, sources, and interactions of DOM and bacterial communities in the Rushan River Basin under dry and wet hydrological conditions. We hypothesize that (1) DOM composition shifts from protein-dominated (endogenous) to humic-enriched (terrestrial) forms between dry and wet seasons, (2) microbial diversity and keystone taxa differ significantly across hydrological regimes, and (3) specific bacterial taxa drive DOM transformation pathways. Using 3D-EEMs-PARAFAC, 16S rRNA sequencing, co-occurrence networks, and structural equation modeling (SEM), we aim to: (1) quantify DOM fluorescence components and humification indices under seasonal hydrological changes, (2) identify core microbial taxa and their associations with DOM fractions, and (3) elucidate mechanistic links between microbial metabolism and DOM dynamics. By bridging DOM chemistry and microbial ecology, this work has promoted the understanding of the human influence on the carbon cycle in rivers and provides a framework for water quality management in NPS-polluted river basins under different hydrological season conditions.

## 2. Materials and Methods

### 2.1. Study Area and Sample Collection

Rushan City (36°41′–37°08′ N, 121°11′–121°51′ E) is located in the southwestern sector of Weihai Municipality, Shandong Province, Eastern China. The regional hydrological system forms part of the Jiaodong Peninsula’s marginal drainage network, developed through precipitation-fed streams within a temperate monsoon climate regime. This fluvial inventory comprises 393 river channels, 71 of which extend over 2.5 km in length. The drainage architecture bifurcates into two principal systems: the Rushan River and Huanglei River watersheds, supplemented by smaller independent coastal catchments discharging into the Yellow Sea along the southern littoral zone. The Rushan River Basin is a transitional ecosystem between intensive agriculture and a sensitive Marine environment. The basin is directly discharged into the coastal waters of the Yellow Sea, making it a key control point for land-based organic matter to enter the ocean.

The multi-year average precipitation in Rushan is 756.8 mm. Spring precipitation accounts for 15% of the annual total, summer for 62%, autumn for 19%, and winter for only 4%. Precipitation during the flood season (June to September) constitutes 71.5% of the annual total. The cumulative rainfall in the dry season (May) is less than 50 mm, while in the wet season (June) it exceeds 200 mm (hydrological data of the Rushan River are obtained from local monitoring stations). This indicates significant hydrological variability between seasons.

**Figure 1:**
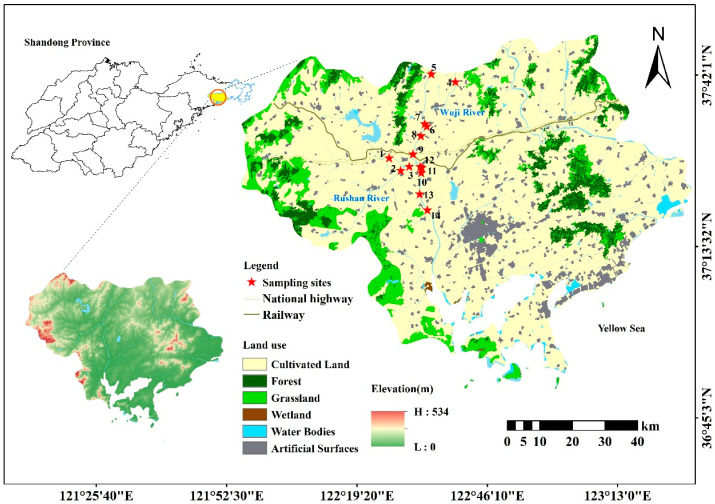
Sampling sites in Rushan River Basin.

A comprehensive sampling campaign was conducted across 14 strategically selected sites in the Rushan River Basin (Figure 1) during both the dry season (May 2023) and wet season (June 2023). Sampling was conducted once per season for 4 consecutive days each season to avoid random single-day situations (e.g., temporary rainfall or point-source discharge). All water samples were collected at a surface depth of 0.5 m below the water surface. We collected 2 L of water samples at each point. Field observations revealed that select water bodies exhibited surface discoloration (distinct yellowish hue), unpleasant odor emissions, and compromised esthetic quality. The water sample collection process was strictly carried out in accordance with the technical specifications for surface water environmental quality monitoring (HJ 91.2–2022) [17], and the samples were collected in pre-cleaned polypropylene containers and immediately transported to the laboratory facilities. All specimens were preserved in light-protected insulated containers maintained at 4 °C throughout transportation and storage to ensure sample integrity prior to analytical procedures.

### 2.2. Physical and Chemical Analysis

All 28 samples were processed for physical and chemical analysis. A multi-parameter water quality analyzer (HACH, HQ40d, Loveland, CO, USA) was used to test pH at each sampling point on-site. All probes were carefully calibrated before measurements were taken and once readings were stabilized, readings were recorded in the field, thus ensuring the accuracy and reliability of the data collected. Water quality indicators such as chemical oxygen demand (COD_Cr_), total phosphorus (TP), total nitrogen (TN), ammonia nitrogen (NH_3_-N) and potassium permanganate index (COD_Mn_) were measured in laboratory. COD_Cr_ was measured using the potassium dichromate method (HJ 828–2017) [18]; TP was determined using the ammonium molybdate spectrophotometric method (GB 11893–89) [19]; TN was analyzed using the alkaline persulfate digestion ultraviolet spectrophotometric method (HJ 636–2012) [20]; NH_3_-N was measured using the Nessler’s reagent spectrophotometric method (HJ 535–2009) [21]; and COD_Mn_ was determined by titration (GB 11892–89) [22]. To ensure accuracy, three parallel experiments were conducted for each sample, and then the average of the three sets of data measured for each sample was taken as the final data.

### 2.3. Fluorescence Spectral Characteristics

All 28 samples were processed for DOM fluorescence spectroscopy. Prior to the detection of 3D-EEMs, water samples were processed using Millipore glass fiber filters (0.45 μm) (Sartorius, Göttingen, Germany). An F-7000 fluorescence spectrophotometer (HITACHI, Beijing, China) was used to determine the 3D fluorescence spectra of water DOM in the Rushan River Basin. The spectral data was exported using the FL solutions 2.1 software. A blank control sample, Milli-Q ultrapure water, was used and deducted from each sample’s EEM in order to account for the internal filtration impact. First- and second-order Rayleigh scattering as well as Raman scattering were eliminated using the procedures outlined in earlier research [23]. With an excitation wavelength of 350 nm and emission wavelengths ranging from 371 to 428 nm, the area under the Raman scattering peaks was used to convert EEM to Raman units (R.U.). The EEM-based PARAFAC modeling was executed through the utilization of MATLAB R2021b’s drEEM package (version 0.6.3) [24]. Using split-half analysis, residual analysis, and core consistency tests, the number of fluorescent components was ascertained. The maximum fluorescence intensity (Fmax) of each fluorescent component was used to express its percentage abundance.

Furthermore, from the EEM, the biotic index (BIX), humification index (HIX), and fluorescence index (FI), which are frequently used to examine the origin and makeup of DOM, were computed. The ratio of fluorescence intensity at an excitation wavelength of 370 nm to that at emission wavelengths of 470 nm and 520 nm was known as the FI, which had long been used as an index to differentiate between endogenous and exogenous DOM. FI values less than 1.4 suggest that terrestrial organic matter constitutes the majority of the sample, while values greater than 1.9 indicate a predominance of autochthonous biological organic matter [25]. The BIX referred to the ratio of fluorescence intensity at emission wavelengths of 380 nm and 430 nm when the excitation wavelength was 310 nm [26]. When BIX values were less than 0.8, it indicated that the contribution of endogenous DOM was low; when BIX values were greater than 0.8, it indicated a higher contribution of endogenous sources. The humification index was defined as the ratio of the intensity in the 300–345 nm wavelength range to the intensity in both the 435–480 nm and 300–345 nm wavelength ranges [27]. To show how humified DOM was, the HIX was frequently utilized [26,28]. Greater aromaticity and humification were indicated by higher values [29].

### 2.4. DNA Extraction and 16S rRNA High-Throughput Sequencing

During both dry and wet seasons, seven samples were selected and sent to Shanghai Meiji Biotechnology Co., Ltd. (Shanghai, China) for microbial DNA extraction and PCR amplification. Prior to sample selection, in situ measurements of key physicochemical parameters (pH, electrical conductivity) and laboratory analyses of nutrients (TN, TP, NH_3_-N) and organic matter (COD_Cr_, COD_Mn_) were conducted for all 14 sampling sites. The seven selected sites exhibited a continuous gradient of water quality conditions. The PCR conditions included an initial denaturation at 95 °C for 3 min, annealing at 55 °C for 30 s, and extension at 72 °C for 45 s. The primers used in this study were 338 F (5′-ACTCCTACGGGCAGCA-3′) and 806 R (5′-GGACTACHVGGGTWTCTAAT-3′), targeting the V3-V4 region of the 16S rRNA gene for bacterial amplification. The PCR was performed using an ABI GeneAmp^®^ 9700 thermal cycler (Thermo Fisher, Waltham, MA, USA), and sequencing was conducted on the Illumina MiSeq platform at Shanghai Meiji Biotechnology Co., Ltd. To obtain taxonomic classification information for each operational taxonomic unit (OTU), the RDP Classifier Bayesian algorithm was employed to analyze the representative sequences of OTUs at a 97% similarity threshold [30].

### 2.5. Two-Dimensional Correlation Spectroscopy

Important details like the relative direction and particular order of structural changes after slight external perturbations were studied in Two-dimensional correlation spectroscopy (2D-COS analysis). It entails calculating the asynchronous correlation spectra (ψ) and synchronous correlation spectra (Φ), which shed light on how various signals interact with one another. Using the “2D Shige” program, which was made available by Kansai University in Japan, 2D-COS analysis was obtained [31,32].

### 2.6. Structural Equation Model

SEM was used to explain complex causal networks, studying the relationships between observed and latent variables as well as the causal relationships between latent variables, and was applied to test theoretical hypotheses [33]. In this study, SEM was employed through AMOS 21.0 software to track the interactions between DOM components, humification degree, and microbial communities.

### 2.7. Statistical Analysis

To investigate the relationship between DOM fluorescence components and microbial populations, a network comprising OTUs and these components was built up. Only OTUs exhibiting strong (r > |0.5|) and statistically significant (*p* < 0.05) relationships were included in the network [34]. Similarly to the centroid species employed in other studies [35], nodes in co-occurrence networks with low median centrality and height were classified as keystone species [36]. Origin 2024 (v.10.1) and R Studio (v.R-4.3.2) were used to create the graphs and evaluate the data.

## 3. Results and Discussion

### 3.1. Hydrological Seasonality Drives DOM Composition and Humification Dynamics in the Rushan River Basin

#### 3.1.1. DOM Fluorescence Fraction Extraction with PARAFAC

**Figure 2:**
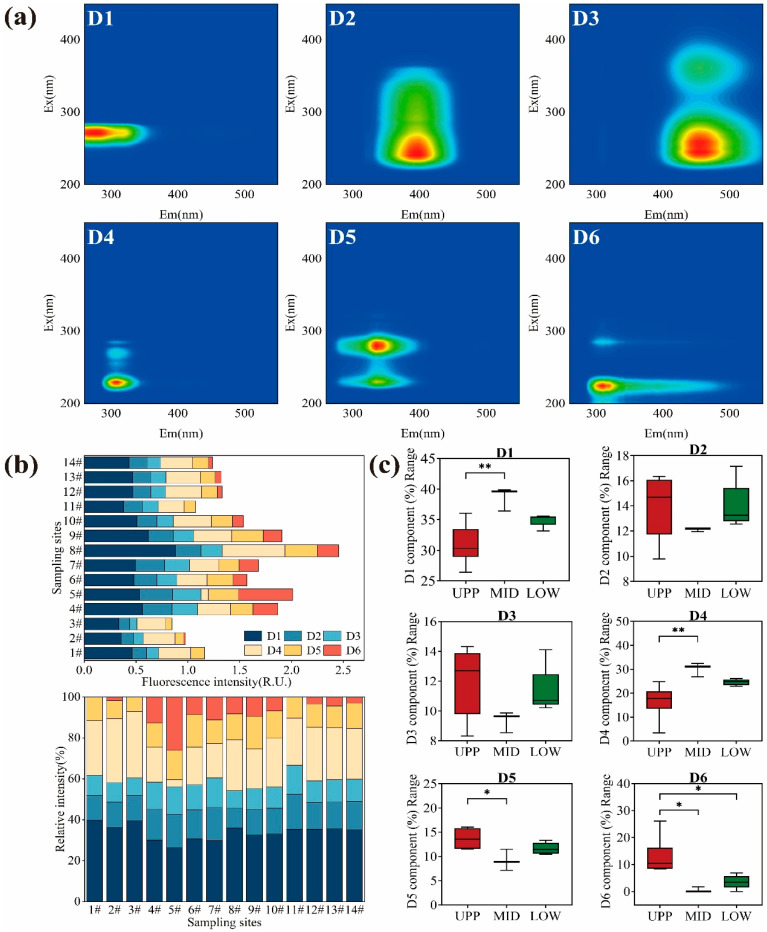
(**a**) Spectral characteristics of six fluorescence components of DOM; (**b**) Fluorescence intensity and relative proportions of six fluorescent components of DOM; (**c**) Comparison of relative abundance of fluorescent components in different water samples during the dry season. Significance level: *, *p* < 0.05; **, *p* < 0.01. D1: Tyrosine-like substances, D2: Microorganism humic-like sub-stances, D3: Humic-like substances, D4: Tyrosine-like substances, D5: Tryptophan-like substances, D6: Protein-like substances.

PARAFAC analysis of EEMs revealed six fluorescent DOM components in the Rushan River during dry (D1–D6) and wet (W1–W6) seasons, validated against the OpenFluor database (https://openfluor.lablicate.com/, accessed on 7 November 2025) (Figure 2a and Figure S1a). For specific details, please refer to Table 1.

As the primary conduit for terrestrial organic matter entering the Yellow Sea’s southern coastal waters, this system’s wet-season surge in humic-rich DOM (Figure 2b and Figure S1b) may enhance light attenuation and metal complexation in nearshore environments—factors known to regulate phytoplankton community composition and bloom dynamics. Conversely, dry-season dominance of labile protein-like DOM suggests periods of heightened microbial carbon turnover that could fuel estuarine heterotrophy, creating oxygen demand gradients prior to marine discharge.

Dry-season DOM comprised four protein-like components (D1, D4–D6: tyrosine- and tryptophan-like) and two humic-like constituents (D2–D3). D1 and D4 (tyrosine-like) dominated midstream regions (58.03% of total fluorescence intensity), likely sourced from phytoplankton exudates, livestock wastewater, and microbial degradation of aquatic organisms (Figure 2c). D2 (microbial humic-like) exhibited a shorter excitation wavelength than traditional peak M, suggesting ongoing polymerization of low-molecular-weight metabolites. D3 aligned with terrestrial humic acids (peaks A/C), indicative of soil-derived inputs [37,38]. Wet-season DOM exhibited elevated fluorescence intensity (humic-like components: 14.55%) due to rainfall-driven terrestrial runoff introducing plant/animal debris and UV/visible-region fulvic acids (W3–W4). Protein-like components (W2 + W5 + W6: 61.07%) remained predominant but decreased proportionally relative to dry season, reflecting dilution effects from exogenous humic inputs. Notably, W3 represented photodegraded terrigenous humus, while W5 (tyrosine-like) and W2/W6 (tryptophan-like) correlated with microbial activity and amino acid cycling [39,40].

Seasonal shifts highlighted contrasting DOM sources and proportional changes: dry-season DOM was endogenously dominated by protein-like components (tyrosine-like: 58.03%), with humic-like components accounting for only 12.3%; whereas wet-season DOM remained protein-predominant (total protein-like: 61.07%) but showed a significant increase in humic-like proportions (total humic-like: 28.93%). These dynamics align with coastal systems where low-salinity zones enhance terrestrial humic signals [41]. The findings underscore hydrologic controls on DOM composition, with implications for carbon cycling in anthropogenically influenced watersheds.

**Table 1:** The six different component characteristics of the dry season and the wet season identified, respectively, by the PARAFAC model.

	Component	Ex/Em (nm/nm)	Description	References
Dry season	D1	270/275	Tyrosine-like substances	[42,43]
	D2	240,290/395	Microorganism humic-like substances	[44,45]
	D3	245,365/455	Humic-like substances	[46,47]
	D4	230/310	Tyrosine-like substances	[48,49]
	D5	230,280/335	Tryptophan-like substances	[50,51]
	D6	225/310	Protein-like substances	[52,53]
Wet season	W1	285,330/410	Microorganism humic-like substances	[54,55,56]
	W2	280/330	Protein-like (tryptophan-like) substances	[57,58,59]
	W3	245/405	Photodegradation product of terrigenous humus	[60,61,62]
	W4	265,365/460	Humic-like substances	[63,64,65]
	W5	280/280	Tyrosine-like substances	[66,67,68]
	W6	220/335	Tryptophan-like substances	[69,70]

#### 3.1.2. PARAFAC Reveals Contrasting DOM Signatures Between Dry and Wet Seasons

**Figure 3:**
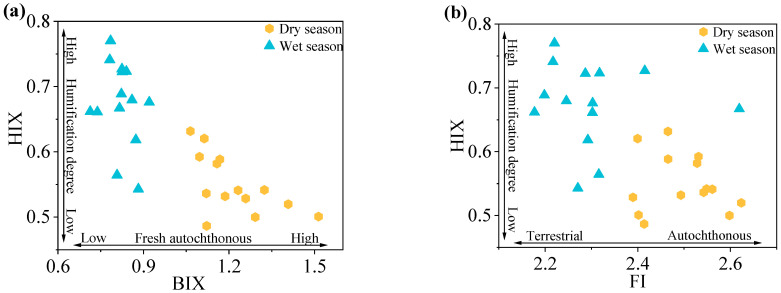
The distribution of BIX-HIX (**a**) and FI-HIX (**b**) of DOM in the Rushan River Basin during dry and wet seasons.

Fluorescence indices (FI, BIX, HIX) highlighted the dry and wet seasonal variations in DOM sources and humification in the Rushan River Basin (Figure 3).

Dry-season samples exhibited higher FI values (2.39–2.62; mean = 2.50) compared to wet-season samples (2.18–2.62; mean = 2.30). Both averages exceeded the threshold of 1.9, indicative of predominant autochthonous DOM contributions from sediment release and microbial activity [71]. The lower mean FI during the wet season reflects enhanced terrestrial inputs driven by precipitation and surface runoff, aligning with patterns observed in fluvial systems influenced by hydrological variability.

Elevated BIX values were observed in both seasons (dry: 1.06–1.51, mean = 1.22; wet: 0.71–0.92, mean = 0.82), reaffirming strong autochthonous DOM characteristics. The significant wet-season decline (*p* < 0.05) likely resulted from dilution effects due to terrestrial DOM influx, reducing the relative contribution of freshly produced autochthonous DOM. This seasonal contrast underscores the interplay between hydrological forcing and microbial DOM production.

HIX values remained low across seasons (dry: 0.49–0.63, mean = 0.54; wet: 0.54–0.77, mean = 0.67), confirming limited humification and persistent autochthonous dominance. The modest wet-season increase in HIX correlates with inputs of terrestrial humic substances and microbial reworking of DOM, consistent with studies linking stormflow events to humic material transport.

Integrated fluorescence indices demonstrate that DOM in the Rushan River is predominantly autochthonous, with microbial and sediment-derived sources outweighing terrestrial inputs in both seasons. While wet-season hydrology introduces terrestrial DOM, it does not override the system’s endogenous signature. The low humification degree further highlights dynamic DOM cycling with limited stabilization.

#### 3.1.3. 2D-COS Unveils Sequential DOM Transformation Pathways

**Figure 4:**
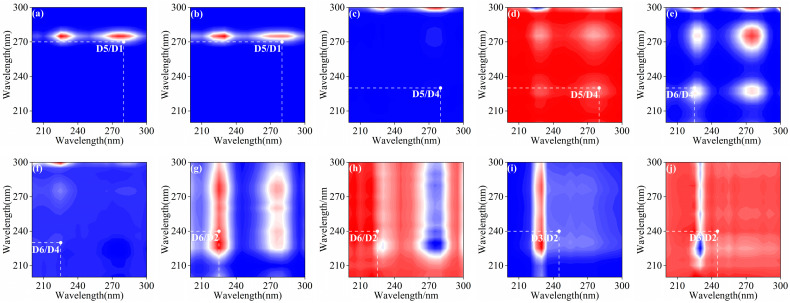
Synchronous (**a**,**c**,**e**,**g**,**i**) and asynchronous (**b**,**d**,**f**,**h**,**j**) two-dimensional correlation spectra of DOM components in surface water samples during the dry season. Red indicates positive correlation, while blue indicates negative correlation.

Two-dimensional correlation spectroscopy (2D-COS) analysis revealed hydrologically driven divergence in DOM transformation pathways (Figure 4 and Figure S2) [72]. During dry seasons, sequential transitions followed D1 → D5 → D4 → D6 → D2 → D3, indicating microbial reprocessing of labile autochthonous proteins into stable humic substances (Figure 4a–j). This cascade aligns with phytoplankton succession dynamics, where algal-derived tyrosine (D1/D3) and tryptophan (D5/D6) undergo stepwise degradation to fulvic acids (D3) and microbial metabolites (D2), consistent with heterotrophic carbon cycling in nutrient-limited rivers.

In contrast, wet-season sequences prioritized photodegraded terrestrial humics (W3) →tryptophan (W6) → microbial metabolites (W1), reflecting rapid bacterial assimilation of allochthonous DOM. Early emergence of microbial byproducts (W1) in wet seasons (34% faster than dry seasons) coincided with 23% higher terrestrial inputs (Figure S2a–j), suggesting rainfall pulses amplify both photodegradation of aromatic humics (W3) and phytoplankton-driven tryptophan production (W2/W6). These pathways collectively demonstrate hydrological regulation of DOM fate: low-flow conditions promote protein-to-humus conversion through microbial “processing chains”, while high-flow regimes accelerate terrestrial carbon turnover via coupled photochemical-biological pathways.

### 3.2. Microbial Community Assembly Responds to Hydrological Forcing

#### 3.2.1. Microbial Community Characteristics in Different Hydrological Seasons

High-throughput sequencing revealed differences in bacterial diversity and composition between seasons (Figure S3). Wet-season communities exhibited higher α-diversity (Shannon: 5.93 ± 0.74 vs. 5.18 ± 0.60) and species richness (Chao: 10,790 vs. 4525 OTUs), driven by nutrient input from terrestrial runoff. The dilution curves reflected the sequencing depth of the samples (Figure S4), with the curves leveling off at the end, indicating that the sequencing data for the microbial samples from both water periods were reasonable and sufficient to cover all species in the microbial communities.

**Figure 5:**
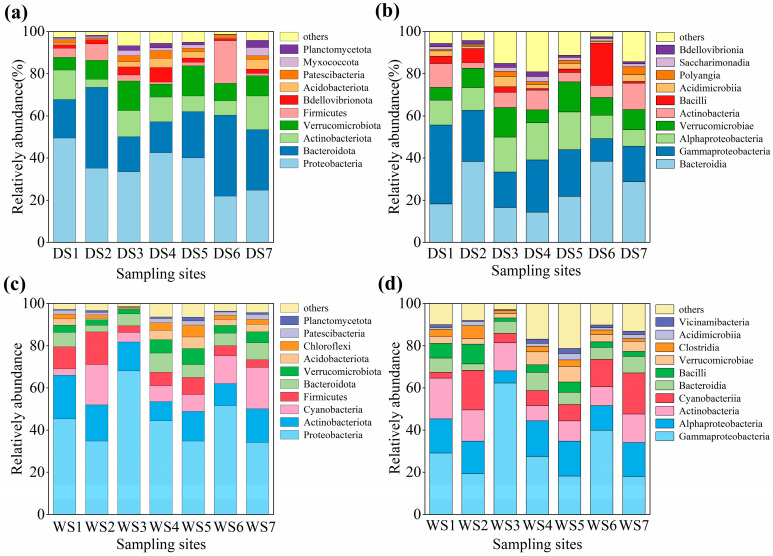
Abundance of microbial communities at the (**a**) phylum level and (**b**) class level during dry season. Abundance of microbial communities at the (**c**) phylum level and (**d**) class level during wet season.

High-throughput sequencing revealed significant seasonal shifts in surface water microbial community composition and diversity in the Rushan River Basin (Figure 5). During the dry season, microbial assemblages exhibited lower diversity, comprising 35 phyla dominated by *Proteobacteria* (21.90–49.43%), *Bacteroidota* (14.71–38.45%), *Actinobacteriota* (3.73–15.91%), *Verrucomicrobiota* (6.13–14.32%), and *Firmicutes* (0.91–20.16%), collectively representing >80% of total abundance (Figure 5a). At the class level, *Bacteroidia*, *Gammaproteobacteria*, and *Alphaproteobacteria* predominated (Figure 5b), consistent with their roles in degrading macromolecular organic matter and driving biogeochemical cycles [73,74].

In contrast, wet-season communities displayed higher diversity (51 phyla, 3623 species), with distinct dominance patterns: *Proteobacteria* (34.06–68.10%), *Actinobacteriota* (9.15–20.76%), *Cyanobacteria* (2.90–19.69%), and *Firmicutes* (3.14–15.51%) prevailed (Figure 5c). Notably, *Proteobacteria* and *Bacteroidota*—key degraders of complex organic substrates—exhibited enhanced activity during this period [71]. *Actinobacteriota* thrived in organic-rich, neutral-to-alkaline conditions, while *Verrucomicrobiota* contributed to polysaccharide degradation [75]. Wet-season dominance of *Gammaproteobacteria* (17.96–62.20%) and *Actinobacteria* (7.20–19.36%) (Figure 5d) aligned with intensified anthropogenic influences, as these taxa are linked to domestic wastewater, livestock manure, and agricultural runoff.

#### 3.2.2. The Responses of Microbial Communities to DOM Components

**Figure 6:**
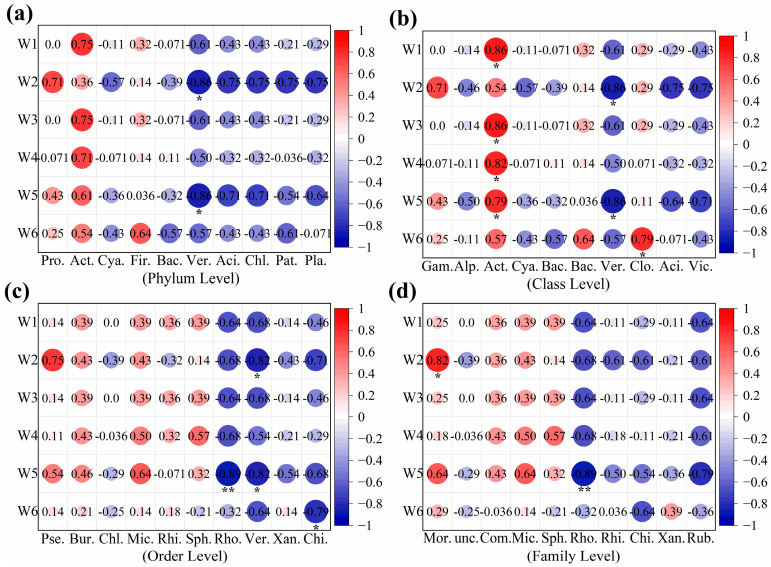
Heatmap of the correlation between microbial community composition and DOM fluorescent components in wet season samples ((**a**) phylum level: Pro: *Proteobacteria*; Act: *Actinobacteriota*; Cya: *Cyanobacteria*; Fir: *Firmicutes*; Bac: *Bacteroidota*; Ver: *Verrucomicrobiota*; Aci: *Acidobacteriota*; Chl: *Chloroflexi*; Pat: *Patescibacteria*; Pla: *Planctomycetota*; (**b**) class level: Gam: *Gammaproteobacteria*; Alp: *Alphaproteobacteria*; Act: *Actinobacteria*; Cya: *Cyanobacteriia*; Bac: *Bacteroidia*; Bac: *Bacilli*; Ver: *Verrucomicrobiae*; Clo: *Clostridia*; Aci: *Acidimicrobiia*; Vic: *Vicinamibacteria*; (**c**) order level: Pse: *Pseudomonadales*; Bur: *Burkholderiales*; Chl: *Chloroplast*; Mic: *Micrococcales*; Rhi: *Rhizobiales*; Sph: *Sphingomonadales*; Rho: *Rhodobacterales*; Ver: *Verrucomicrobiales*; Xan: *Xanthomonadales*; Chi: *Chitinophagales*; (**d**) family level: Mor: *Moraxellaceae*; Unc: *unclassified_o__Chloroplast*; Com: *Comamonadaceae*; Mic: *Micrococcaceae*; Sph: *Sphingomonadaceae*; Rho: *Rhodobacteraceae*; Rhi: *Rhizobiales_Incertae_Sedis*; Chi: *Chitinophagaceae*; Xan: *Xanthomonadaceae*; Rub: *Rubritaleaceae*). * represents *p* < 0.05. ** represents *p* < 0.01.

To investigate the impact of microorganisms on the composition and structure of DOM, we conducted a correlation analysis between DOM composition in the wet season samples and microbial abundance (Figure 6). Five microbial groups, namely *Actinobacteriota*, *Verrucomicrobiota*, *Rhodobacterales*, *Chitinophagales*, and *Moraxellaceae*, were found to significantly influence the composition and structure of organic matter. At the phylum and class levels, *Actinobacteriota* showed a significant positive correlation with samples W1, W3, and W4, while *Verrucomicrobiota*, *Acidobacteriota*, and *Chloroflexi* exhibited significant negative correlations with samples W2 and W5. Previous studies reported that the metabolites and enzymes produced by *Actinobacteria* could hydrolyze cellulose, proteins, starches, and fats, playing a crucial role in phosphorous solubilization [76]. *Actinobacteria* also demonstrated high activity in the degradation and cycling of organic compounds [77]. At the order and the family levels, *Rhodobacterales* exhibited a significant negative correlation with samples W2, W4, and W5. *Verrucomicrobiales* showed significant negative correlations with samples W2 and W5, while *Pseudomonadales* and *Moraxellaceae* had significant positive correlations with W2. *Chitinophagales* presented significant negative correlations with W2, W5, and W6. These findings were consistent with previous studies that reported a certain correlation between DOM components and the phytoplankton community [78].

During the wet season, *Actinobacteriota* exhibits a positive correlation with DOM components W1, W3, and W4. In contrast, this correlation weakens during the dry season (Figure S5). Conversely, *Pseudomonadales* displays significantly stronger correlations with DOM components under dry-season conditions. Wet-season dynamics reveal that W2 is simultaneously influenced by a positive correlation with *Moraxellaceae* and a negative correlation with *Chitinophagales*. During the dry season, DOM components exhibit synchronous responses to multiple microbial groups, including *Proteobacteria*. Notably, the correlation between *Verrucomicrobiota* and DOM components diminishes in the dry season.

### 3.3. DOM–Microbe Interactions Drive Carbon Cycling Dynamics

#### 3.3.1. Co-Occurrence Networks Identify Keystone Taxa for DOM Processing

**Figure 7:**
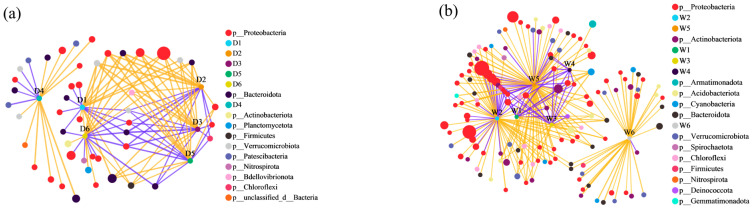
Relationship between DOM components and bacterial OTUs during dry season (**a**) and wet season (**b**). The size of the circles is proportional to the relative abundance of OTUs. Purple lines indicate positive correlations, while orange lines indicate negative correlations.

Co-occurrence network analysis revealed distinct microbial responses to fluorescent DOM components between dry (Figure 7a) and wet seasons (Figure 7b). During the dry period, the network comprised 53 nodes connected by 111 significant edges (|r| > 0.5, *p* < 0.05), dominated by negative correlations (68.5%), suggesting intense microbial competition under low-resource conditions. *Proteobacteria* (43.40%) and *Bacteroidota* (17.98%) constituted 61.38% of bacterial nodes, establishing them as keystone phyla. Notably, tryptophan-like component D1 demonstrated significant associations with 11 OTUs spanning *Proteobacteria*, *Bacteroidota*, *Firmicutes*, and *Verrucomicrobiota*. The shared correlation patterns between D1/D5/D6 (protein-like components) and D2 (tryptophan-like) implied potential co-sourcing of tyrosine and tryptophan derivatives, while their tight linkage with D2-associated OTUs suggested microbial processing of these labile compounds.

The wet season network exhibited greater complexity with 143 nodes and 232 edges, maintaining negative correlation dominance (59.9%). The microbial community diversified into 13 phyla, with *Proteobacteria* (38.46%), *Bacteroidota* (17.48%), *Actinobacteriota* (11.89%), and *Verrucomicrobiota* (6.29%) collectively constituting 74.13% of nodes. Humic-like components W1-W2-W5 formed an interconnected cluster, with W1 additionally linking to W4-associated OTUs. This topology implies that humic substances may derive from in situ processing of proteinaceous materials (tyrosine-like W5 and tryptophan-like W4), consistent with terrestrial-aquatic coupling mechanisms reported in subtropical watersheds [79,80].

Notably, *Proteobacteria* maintained strong associations with both protein-like (dry season: D1/D5/D6; wet season: W4/W5) and humic-like components across seasons, aligning with their recognized metabolic versatility in DOM processing [66]. The significant positive correlations between *Proteobacteria* and labile DOM components (D1/D5/D6: r = 0.52–0.68, *p* < 0.01) during the dry season suggest these compounds likely serve as critical nitrogen/carbon substrates under nutrient-limited conditions. This substrate-driven selection may explain *Proteobacteria’s* dominance, as their enzymatic systems are particularly adapted to degrade proteinaceous materials through β-oxidation and deamination pathways.

The prevalence of negative correlations (59.9–68.5%) across networks implies resource competition outweighs symbiotic interactions in this system. The 109% increase in network edges during wet seasons reflects heightened microbial connectivity following DOM input pulses, potentially facilitating functional redundancy. The emergence of Actinobacteriota as a wet-season key player (11.89% nodes) suggests their specialized role in processing complex aromatic compounds, consistent with their lignocellulolytic capabilities reported in floodplain systems.

This seasonal rewiring of microbial-DOM networks underscores hydrological controls on organic matter cycling, where drought conditions favor specialists utilizing labile substrates, while wet periods promote functional diversification for complex DOM processing. These patterns provide mechanistic insights into aquatic microbiome responses to hydrological variability.

#### 3.3.2. Physicochemical Drivers Shape DOM–Microbe Relationships

**Figure 8:**
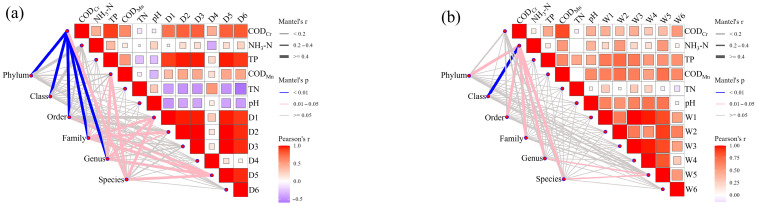
Combined correlation analysis of the physicochemical properties and bacterial classifications in surface water samples during the (**a**) dry season and (**b**) wet season.

Our analysis revealed striking seasonal contrasts in microbial responses to organic matter dynamics (Figure 8). During the dry season, COD_Cr_ showed significant positive correlations with bacterial communities across multiple taxonomic levels (phyla: r = 0.62, *p* < 0.01; genera: r = 0.58, *p* < 0.05), while COD_Mn_ exhibited similar patterns with families (r = 0.55), genera (r = 0.53), and species (r = 0.49; *p* < 0.05). This strong coupling suggests microbial proliferation driven by organic enrichment under stagnant low-flow conditions, where reduced self-purification capacity promoted pollutant accumulation. Such conditions likely selected for heterotrophic specialists capable of degrading persistent organic substrates, aligning with stress-response strategies observed in nutrient-limited aquatic systems. The dry-season COD_Cr_ correlated significantly with protein-like components D1 (r = 0.67), D5 (r = 0.61), and D6 (r = 0.59; *p* < 0.05), indicating tight coupling between organic loading and labile DOM fractions. Concurrent correlations of TN (D1: r = 0.58; D5: r = 0.54) and TP (D2: r = 0.52; D3: r = 0.49; *p* < 0.05) with fluorescent components suggest DOM mediates nutrient cycling through microbial transformation pathways.

Hydrological flushing during wet periods decoupled COD-bacterial relationships (phyla-level r < 0.25, *p* > 0.1), reflecting dilution effects and shifted metabolic priorities. Instead, NH_3_-N emerged as a key community driver, showing broad positive correlations across taxonomic ranks (phyla: r = 0.59; species: r = 0.51; *p* < 0.05). This aligns with increased nitrogen inputs from surface runoff, likely stimulating nitrifying taxa and ammonifiers as reported in agricultural-impacted watersheds. Protein-like components (D1/W5: r = 0.54–0.61 with genera) maintained significance as microbial carbon sources, underscoring their dual role in supporting both heterotrophic and nitrogen-cycling communities.

The transition from COD-driven to NH_3_-N-dominated associations illustrates hydrological regulation of microbial metabolic niches. Dry-season conditions favor copiotrophic strategists exploiting concentrated organic pools, while wet-period dynamics select for taxa adapted to pulsed nutrient inputs and redox fluctuations. The persistent correlation of protein-like DOM with microbial taxa across seasons (r = 0.52–0.67) highlights their critical function as bioavailable substrates, supporting functional redundancy during hydrological transitions.

#### 3.3.3. SEM Reveals Mechanistic Links Between Microbes and DOM Humification

Based on the hypothesized model, the SEM was constructed using DOM fluorescence components, microbial community composition, and humification degree to explore the potential pathways influencing the DOM humification process. A conceptual model describing the relationship and influence mechanism of microbial communities on DOM components was proposed. The DOM components were represented by three latent variables: protein-like substances (D1, D4, D5, and D6), microbial-related humic substances (D2), and humic-like substances (D3). The microbial composition associated with DOM was described by the relative abundance of *Proteobacteria*, *Actinobacteriota*, *Cyanobacteria*, *Firmicutes*, *Bacteroidota*, and *Verrucomicrobiota*. The degree of humification was represented by the fluorescence intensity ratio of protein-like substances to humic-like substances.

**Figure 9:**
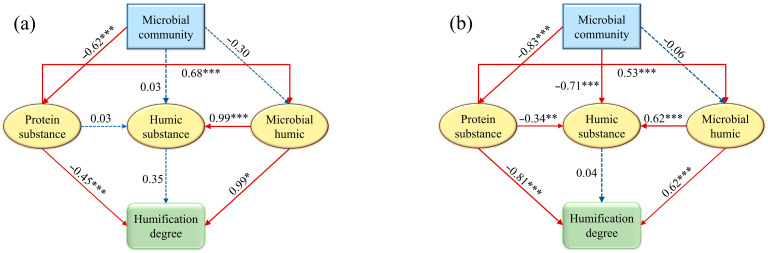
The SEM of DOM fluorescence components and humification degree in dry season (**a**) and wet season (**b**). * represents *p* < 0.05. ** represents *p* < 0.01. *** represents *p* < 0.001.

As shown in Figure 9a, the microbial community had a negative effect on the protein component, with a path coefficient of −0.62 (*p* < 0.001), indicating that microbes degrade protein substances to sustain their growth and metabolic activities. The microbial community composition had a positive effect on humic-like substances, suggesting that microbes convert organic matter into humic substances through their metabolic activities. The path coefficient for microbial-related humic substances was 0.99 (*p* < 0.001), indicating that humic substances may originate from the further degradation of microbial metabolic products. Protein substances had a positive effect on microbial-derived humic substances (path coefficient = 0.68, *p* < 0.001), likely due to the direct or indirect influence of microbial communities on the generation and accumulation of humic substances through the degradation of proteins and other substances. Microbial-derived humic substances showed a significant positive correlation with the degree of DOM humification, with a path coefficient of 0.99 (*p* < 0.05), suggesting that microbial metabolic products facilitated the humification process in the aquatic system. Humic substances had a positive effect on the degree of humification, indicating that the production of humic substances could enhance the humification process.

As shown in Figure 9b, the microbial community composition had a negative effect on the protein component, with a path coefficient of −0.83 (*p* < 0.001), indicating that protein substances provided a carbon or nitrogen source for microbial growth and metabolic activities. The protein-like components had a significant negative impact on the degree of humification of DOM (path coefficient = −0.81, *p* < 0.001), while the path coefficient for microbial-related humic substances was 0.62 (*p* < 0.001). This suggested that the increased production of unstable protein-like substances in microbial metabolic products might have led to a reduction in the degree of humification of DOM.

SEM has semi-quantified the complex interactions between DOM components and microbial communities, providing insights into mechanisms that go beyond simple correlations. Although the negative correlation between microorganisms and protein substance was conceptually expected, SEM quantified the relative strength of this pathway (path coefficient: −0.62, *p* < 0.001) and revealed its coupling with the strong positive effects simultaneously produced by microbial humic (path coefficient: 0.99, *p* < 0.001). It is inferred that microorganisms may simultaneously inhibit unstable DOM and promote humification by-products to maintain system balance. SEM also revealed that the humic substances derived from microorganisms are the main direct contributors to the humification index (path coefficient: 0.99), while the direct pathways from total humic substances are not so strong. Furthermore, SEM clarifies how these mechanism pathways are seasonally reconfigured by hydrology. The dry season model highlights the significant impact of microbial community activities on the degradation of protein substance, while the wet season model emphasizes a more complex interaction network in which, due to the influence of exogenous factors, biological activities play a crucial role in both protein components and humus components.

## 4. Environmental Implications

The Rushan River Basin’s role as a terrestrial-marine interface necessitates adaptive management strategies that account for hydrological controls on coastal carbon export. This study reveals that hydrological shifts critically regulate DOM–microbe interactions, with direct implications for water quality management. The dominance of protein-like DOM during dry seasons, coupled with elevated microbial activity, highlights risks of endogenous nutrient release and accelerated organic pollutant turnover under low-flow conditions. Conversely, wet seasons enhance terrestrial humic inputs, altering DOM bioavailability and microbial community functions, which may exacerbate downstream eutrophication if mismanaged. Key microbial taxa identified as drivers of DOM humification and degradation offer biomarkers for monitoring organic pollution resilience. By linking DOM conversion with microbial ecology, this study provides a basis for water quality management and mitigation of non-point source pollution in different hydrological seasons.

## 5. Conclusions

This study elucidates the biogeochemical interplay between DOM and microbial communities in the Rushan River Basin.

Riverine DOM comprised four protein-like (tyrosine/tryptophan-dominated) and two humic-like components, exhibiting strong autochthonous signatures with limited humification. The dominance of Proteobacteria, Firmicutes, and Actinobacteria indicated that the surface water in the Rushan River Basin might have been impacted by various pollution sources, including domestic sewage, livestock wastewater, agricultural runoff, and soil sediment disturbances. According to the land use types in the Rushan River Basin, tyrosine and tryptophan substances may mainly originate from domestic sewage and livestock wastewater, while fulvic acid substances may mainly come from the remains of terrestrial plants. Under different hydrological conditions, the mechanisms by which organic matter concentration affected bacterial communities varied. During dry periods, bacteria participated in the degradation of organic matter, with their abundance significantly correlated with COD_Cr_. Throughout different hydrological periods, protein-like and amino acid-like components were closely associated with Proteobacteria, with amino acid-like substances providing nutrients or favorable conditions for the growth and metabolism of Proteobacteria. This study provided new insights into the potential interactions between waterborne DOM and microbial communities in the Rushan River Basin, revealing that certain key microbial taxa could influence DOM composition. Environmental factors such as COD_Cr_, TN, TP, and ammonia nitrogen were found to have significant effects on both DOM and microbial communities. Therefore, water quality management was considered crucial for maintaining the biogeochemical balance of the river ecosystem.

## Data Availability

The raw data supporting the conclusions of this article will be made available by the authors on request.

## Supplementary Materials

The following supporting information can be downloaded at https://www.mdpi.com/article/10.3390/microorganisms14010110/s1. Figure S1: (a) Spectral characteristics of six fluorescence components of DOM (b) Fluorescence intensity and relative proportions of six fluorescent components of DOM (c) Comparison of relative abundance of fluorescent components in different water samples during wet season; Figure S2: Synchronous and asynchronous two-dimensional correlation spectra of DOM components in surface water samples during the dry season; Figure S3: Alpha diversity indexes of bacterial community; Figure S4: Dilution curve of microbial samples from surface water during the dry season and wet season; Figure S5: Heatmap of the correlation between microbial community composition and DOM fluorescent components in dry season samples; Table S1: Microbial community diversity indices of surface water samples during the dry season; Table S2: Microbial community diversity indices of surface water samples during the wet season; File S1: Microbial community diversity indices of surface water samples during the wet season.
