# Supplementary material for: Hydrological Seasonality Drives DOM–Bacteria Interactions in the Rushan River Basin
Source: Microorganisms. 2026 Jan 5;14(1):110. doi: 10.3390/microorganisms14010110 (PMC12843783; doi:10.3390/microorganisms14010110)
Supplement: Supplementary file 1 [file microorganisms-14-00110-s001.zip › microorganisms-3925671-supplementary.pdf]

Supporting Information

Interaction characteristics of dissolved organic matter and bacterial communities in the Rushan river basin under different hydrological scenarios.

Shanshan Zheng [1,2,†], Fan Feng [1,†], Dongping Liu [1], Feng Qian [1,*], Xiaolin Xie [1], Huibin Yu [1] and Yonghui Song [1]

[1] State Key Laboratory of Environmental Criteria and Risk Assessment, Chinese Research Academy of Environmental Sciences, Beijing 100012, China; m18661373270@163.com (S.Z.); qbunny1634@163.com (F.F.); 18339916386@163.com (D.L.); xxlin0108@163.com (X.X.); yhbybx@163.com (H.Y.); songyh@craes.org.cn (Y.S.)

[2] College of Environment, Liaoning University, Shenyang 110036, China

* Correspondence: qianfeng@craes.org.cn

† These authors contributed equally to this work.

SUPPORTING INFORMATION CONTENT:

The supporting information contains 8 pages with 5 figures and 2 tables.

Figure S1. (a) Spectral characteristics of six fluorescence components of DOM (b) Fluorescence intensity and relative proportions of six fluorescent components of DOM (c) Comparison of relative abundance of fluorescent components in different water samples during wet season. Significance level: *, p<0.05; **, p<0.01. 4

Figure S2. Synchronously and asynchronously plotted 2D-COS of DOM components in surface water samples during the wet season. red indicates positive correlation, while blue indicates negative correlation. 5

Figure S3. Alpha diversity indexes of bacterial community (a: Chao index; b: Shannon index)......6

Figure S4. Dilution curve of microbial samples from surface water during the (a) dry season and (b) wet season. 6

Figure S5. Heatmap of the correlation between microbial community composition and DOM fluorescent components in dry season samples(a: Phylum level; b: Class level; c: order level; d: Family level). 8

Table S1 Microbial community diversity indices of surface water samples during the dry season. .9

Table S2 Microbial community diversity indices of surface water samples during the wet season. 9

Abbreviations .... **Error! Bookmark not defined.**

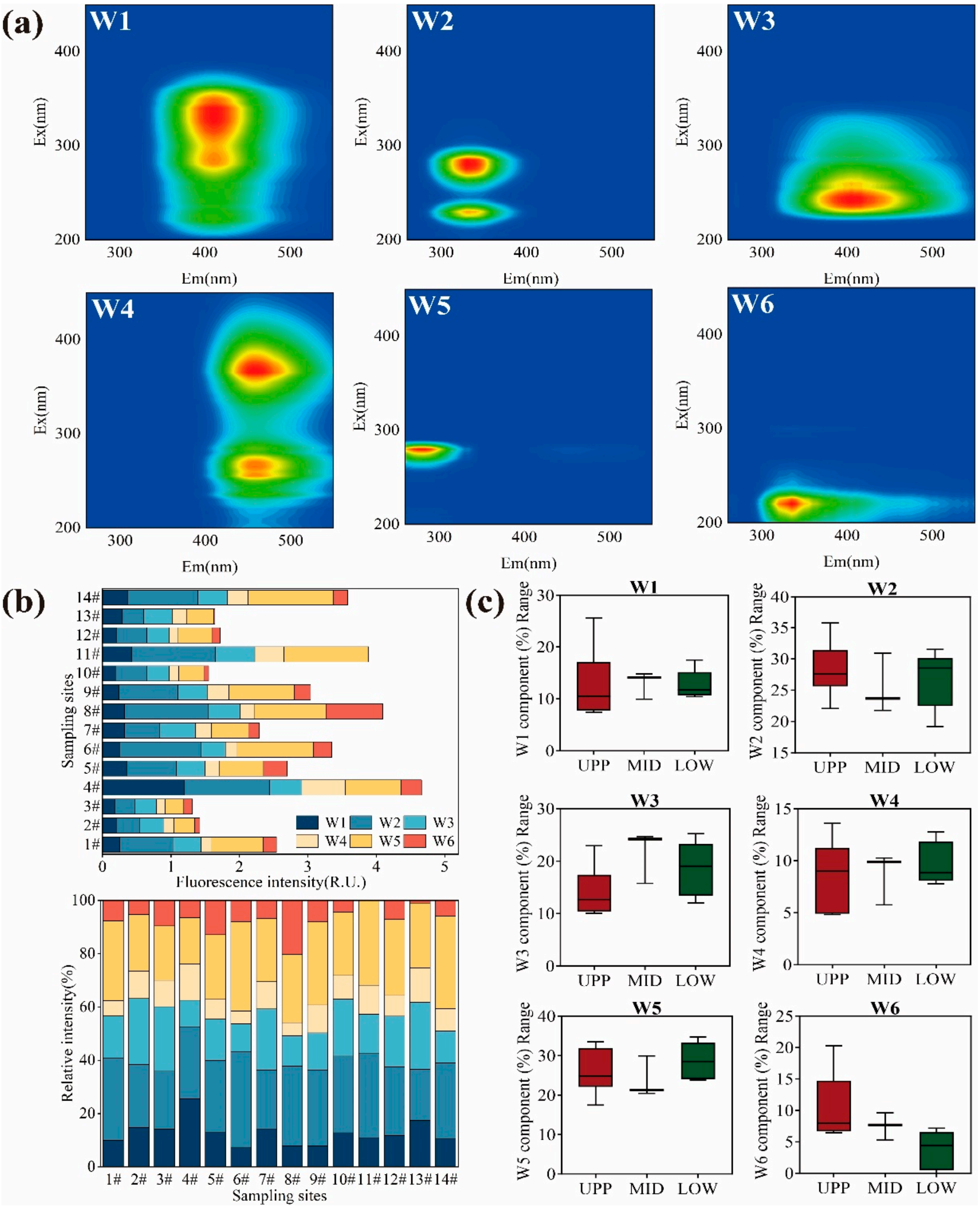

(a)
W1
W2
W3
W4
W5
W6
Ex(nm)
Em(nm)
(b)
Sampling sites
Fluorescence intensity(R.U.)
W1 W2 W3
W4 W5 W6
Relative intensity(%)
Sampling sites
(c)
W1 component (%) Range
W2 component (%) Range
W3 component (%) Range
W4 component (%) Range
W5 component (%) Range
W6 component (%) Range
UPP MID LOW

Figure S1. (a) Spectral characteristics of six fluorescence components of DOM (b) Fluorescence intensity and relative proportions of six fluorescent components of DOM (c) Comparison of relative abundance of fluorescent components in different water samples during wet season. Significance level: *, $p<0.05$; **, $p<0.01$. W1: Microorganism humic-like substances, W2: Protein-like (tryptophan-like) substances, W3: Photodegradation product of terrigenous humus, W4: Humic-like substances, W5: Tyrosine-like substances, W6: Tryptophan-like substances.

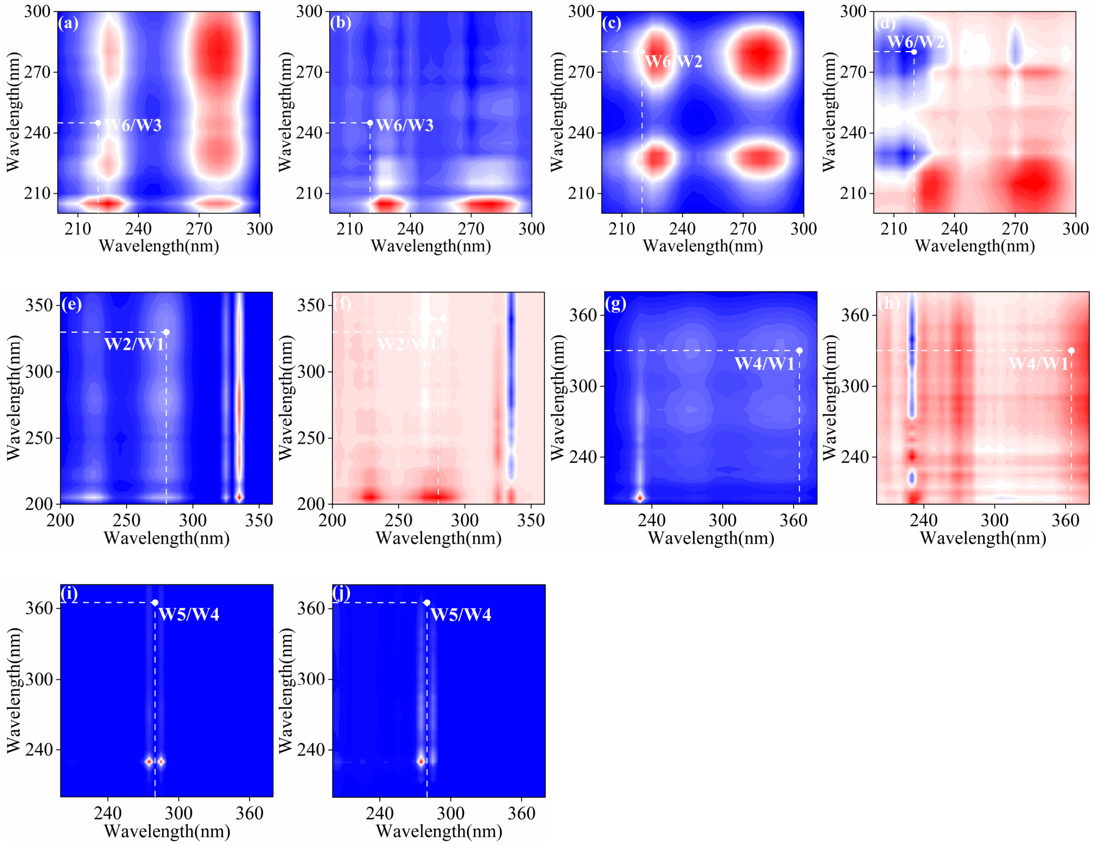

Figure S2. Synchronous (a,c,e,g,i) and asynchronous (b,d,f,h,j) two-dimensional correlation spectra of DOM components in surface water samples during the dry season. Red indicates positive correlation, while blue indicates negative correlation.

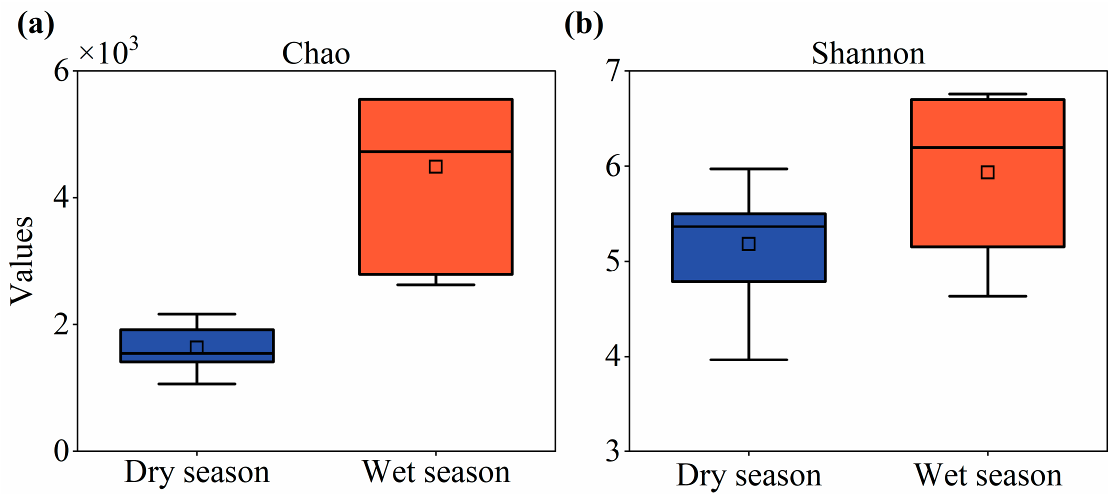

Figure S3. Alpha diversity indexes of bacterial community (a: Chao index; b: Shannon index).

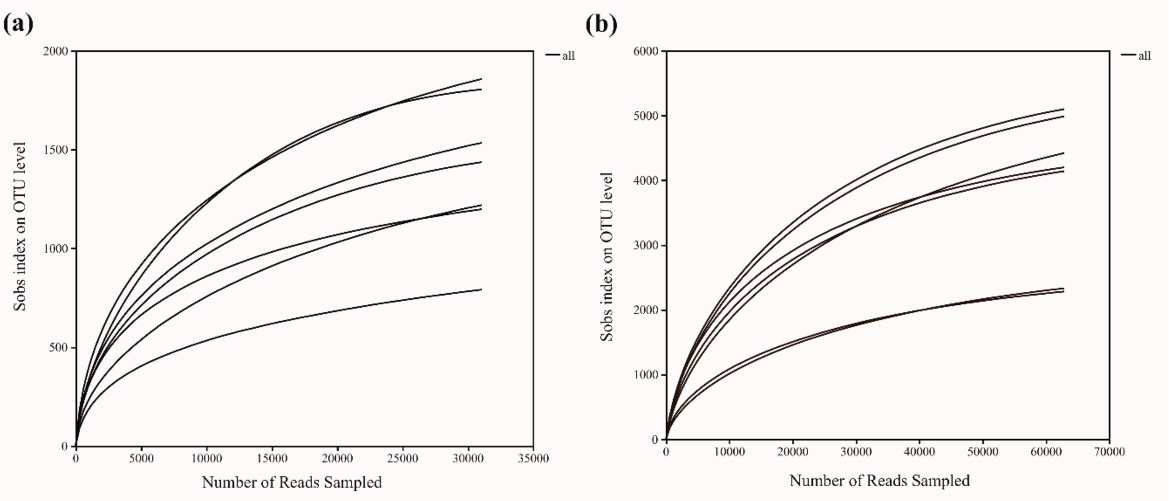

Figure S4. Dilution curve of microbial samples from surface water during the (a) dry season and (b) wet season.

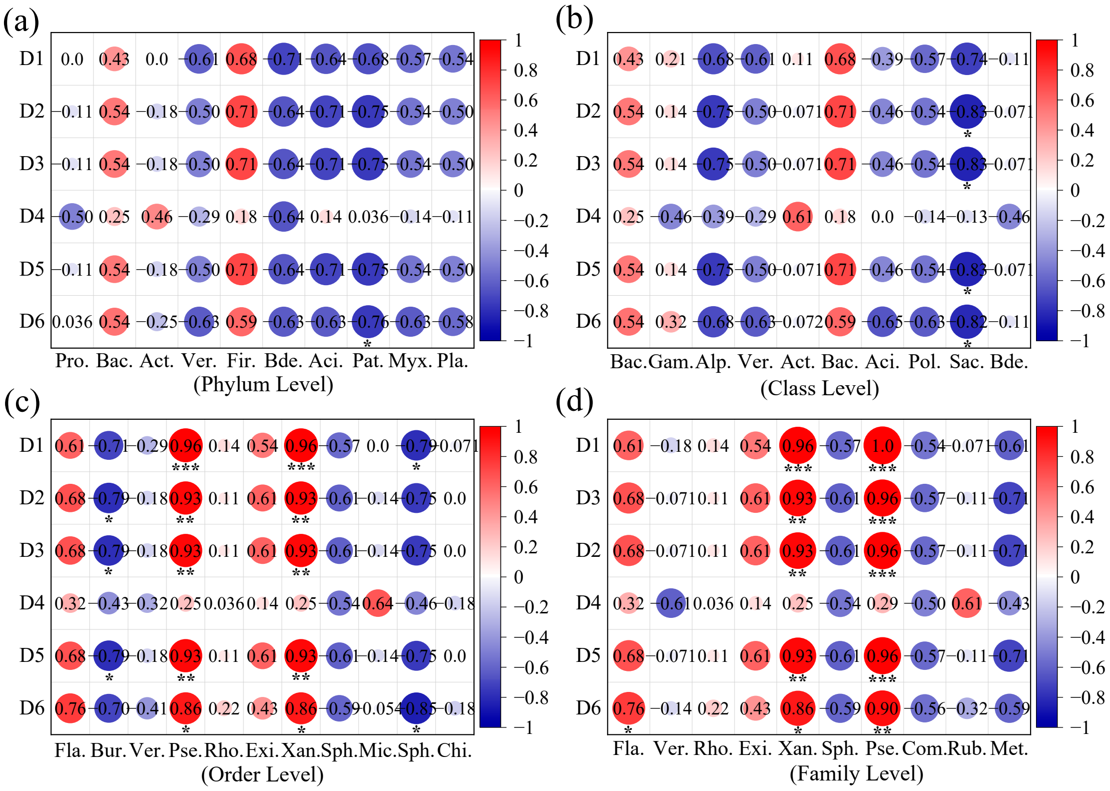

Figure S5. Heatmap of the correlation between microbial community composition and DOM fluorescent components in dry season samples ((a) phylum level: Pro: *Proteobacteria*; Act: *Actinobacteriota*; Cya: *Cyanobacteria*; Fir: *Firmicutes*; Bac: *Bacteroidota*; Ver: *Verrucomicrobiota*; Aci: *Acidobacteriota*; Chl: *Chloroflexi*; Pat: *Patescibacteria*; Pla: *Planctomycetota*; (b) class level: Gam: *Gammaproteobacteria*; Alp: *Alphaproteobacteria*; Act: *Actinobacteria*; Cya: *Cyanobacteriia*; Bac: *Bacteroidia*; Bac: *Bacilli*; Ver: *Verrucomicrobiae*; Clo: *Clostridia*; Aci: *Acidimicrobiia*; Vic: *Vicinamibacteria*; (c) order level: Pse: *Pseudomonadales*; Bur: *Burkhold-eriales*; Chl: *Chloroplast*; Mic: *Micrococcales*; Rhi: *Rhizobiales*; Sph: *Sphingomonadales*; Rho: *Rhodobacterales*; Ver: *Verrucomicrobiales*; Xan: *Xanthomonadales*; Chi: *Chitinophagales*; (d) family level: Mor: *Moraxellaceae*; Unc: *unclassified_o__Chloroplast*; Com: *Comamonadaceae*; Mic: *Micrococcaceae*; Sph: *Sphingomonadaceae*; Rho: *Rhodobacteraceae*; Rhi: *Rhizobiales_Incertae_Sedis*; Chi: *Chitinophagaceae*; Xan: *Xanthomonadaceae*; Rub: *Rubritaleaceae*). * represents $p<0.05$. ** represents $p<0.01$

Table S1 Microbial community diversity indices of surface water samples during the dry season.

Sample\Estimators	ace	chao	sobs	shannon	simpson	coverage
DS1	1897.311	1832.486	1804	5.482056	0.014495	0.993725
DS2	1691.742	1539.059	1219	4.785425	0.023821	0.986581
DS3	2326.104	2167.323	1857	5.970506	0.006825	0.98391
DS4	2004.363	1918.222	1534	5.498258	0.013034	0.985326
DS5	1431.402	1409.193	1198	5.363608	0.01302	0.991086
DS6	1064.634	1062.486	791	3.963461	0.076181	0.992084
DS7	1671.86	1549.438	1436	5.20309	0.025072	0.990218

Table S2 Microbial community diversity indices of surface water samples during the wet season.

Sample\Estimators	ace	chao	sobs	shannon	simpson	coverage
WS1	4823.213	4630.059	4139	6.195407	0.01637	0.985093
WS2	2773.067	2627.713	2281	5.150529	0.026928	0.990709
WS3	2951.037	2789.968	2331	4.632464	0.04765	0.988896
WS4	4804.044	4727.187	4198	6.696994	0.004153	0.985762
WS5	5867.686	5548.604	5097	6.755564	0.004796	0.982373
WS6	5809.665	5550.696	4419	5.77445	0.020425	0.976725
WS7	5885.755	5547.63	4985	6.331947	0.020486	0.980989
